# Acute Myocardial Infarction Due to Microvascular Obstruction in a Young Woman Who Recently Recovered from COVID-19 Infection

**DOI:** 10.3390/jcdd8060066

**Published:** 2021-06-05

**Authors:** Abukar Mohamed Ali, Daanyaal Wasim, Terje H. Larsen, Nigussie Bogale, Øyvind Bleie, Sahrai Saeed

**Affiliations:** Department of Heart Disease, Haukeland University Hospital, 5021 Bergen, Norway; abukar.mohamed.ali@helse-bergen.no (A.M.A.); daanyaal.wasim@helse-bergen.no (D.W.); terje.hjalmar.larsen@helse-bergen.no (T.H.L.); nigussie.bogale@helse-bergen.no (N.B.); oyvind.bleie@helse-bergen.no (Ø.B.)

**Keywords:** COVID-19, case-report, echocardiography, myocardial infarction, strain, cardiac magnetic resonance imaging

## Abstract

Although cardiovascular complications are common in hospitalized COVID-19 patients, those with milder cases who recovered at home are less studied. Here, we report the case of a young woman who recently recovered from COVID-19 at home. A week after recovery, she was admitted to our institution with acute chest pain, signs of ischemia on the electrocardiogram and elevated cardiac troponins. Coronary angiography showed normal epicardial coronary arteries, but the cardiac magnetic resonance showed transmural late gadolinium enhancement (LGE) in the mid-ventricular level of the lateral wall. The findings were strongly suggestive of a minor transmural myocardial infarction. This case report highlights the role of multimodality imaging in detecting cardiac injury in COVID-19 patients as well as the fact that mild COVID-19 cases who recovered at home are also exposed to thromboembolic events during the convalescent period.

## 1. Introduction

Although the majority of patients with coronavirus disease 2019 (COVID-19) experience mild symptoms and recover, more severe forms of the disease may lead to acute respiratory distress syndrome and multiple organ failure. The cardiovascular (CV) complications of COVID-19 include myocarditis-like injury, acute coronary syndromes, thromboembolic events and arrhythmias [1].

Cardiac injury in COVID-19 has different manifestations ranging from milder forms of chest pain and slightly elevated cardiac troponins and pro-brain natriuretic peptides (pro-BNP), to more severe forms of acute coronary syndromes (non-ST-segment-elevation myocardial infarction [NSTEMI] and ST-segment-elevation myocardial infarction [STEMI]), myocarditis, pericardial effusion, tamponade, stress-cardiomyopathy, right and left ventricular systolic dysfunction, pulmonary hypertension, cardiogenic shock and death [1,2,3,4,5,6]. Although CV complications are common in hospitalized COVID-19 patients, those who recover at home are less studied. Hence, in the present case study we present the clinical course of acute myocardial infarction in a 55-year-old woman who recently recovered from COVID-19 at home. The focus is directed towards the role of multimodality imaging to reveal cardiac injury in COVID-19.

## 2. Case Presentation

We report the case of a 55-year-old woman with no previous cardiovascular disease or history of smoking. She tested positive for COVID-19 after exhibiting symptoms of fever, severe cough, headache, shortness of breath and lethargy. The patient was exposed to the virus through her spouse who had similar symptoms. She was assessed by her general physician and considered to be clinically stable to self-isolate at home. The fever subsided on day 9, and a repeated COVID-19 polymerase chain reaction (PCR) returned negative on day 13. She resumed work on day 15, despite having mild residual functional dyspnea.

The patient was admitted to our Cardiology Department on day 20 due to acute chest pain radiating to both arms and the back. During examination, the vital signs were normal. The blood pressure was 130/70 mmHg, the oxygen saturation 97% in room air, and the temperature was 37.1 °C. The auscultation of the heart and lungs was unremarkable. There was no murmur or pericardial friction rub. No signs of jugular venous dilatation or peripheral edema were noted.

### Investigations

**Figure 1:**
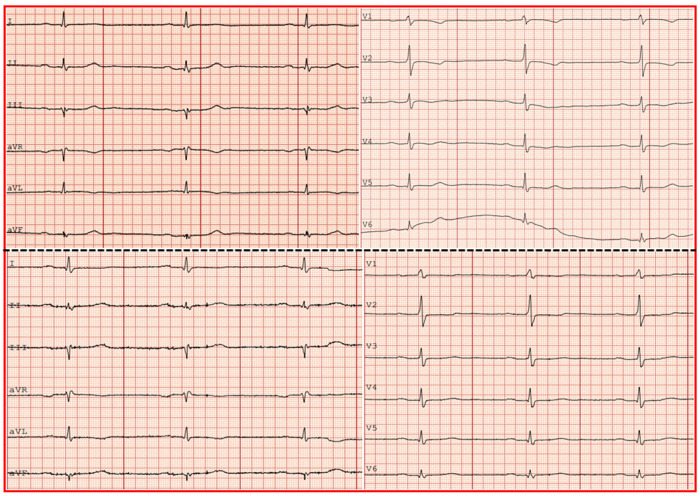
Electrocardiogram on admission (upper panel) showing sinus rhythm and T-wave inversion in leads V1–V4 and AVL. Electrocardiogram at discharge (lower panel) showing sinus rhythm and regression of the T-wave inversion in leads V1–V4.

The electrocardiogram (ECG) showed sinus bradycardia 50 beats per minute (bpm), a normal axis and slight ST segment depression with T-wave inversion in leads V1–V4 and T-wave inversion in AVL (Figure 1, upper panel). 

A bedside echocardiogram performed on admission with a handheld device (V-Scan) showed a normal left ventricular (LV) ejection fraction and no signs of regional wall motion abnormalities or valvular dysfunction. 

The laboratory tests on admission showed a normal hemoglobin of 13.6 g/dL (reference range: 11.0–15.0 g/dL), white blood cell count of 9.7 × 10^9^/L (reference range: 3.5–11.0 × 10^9^) and thrombocytes of 311 × 10^9^/L (reference range: 165–387). The serum ferritin level was increased to 470 μg/L on admission and declined to 380 μg/L at discharge (reference range: 18–240 μg/L). C reactive protein was normal (3 mg/L) on admission but slightly raised to 10 mg/L the following day (reference range: 0–5 mg/L). D-dimer was also slighted elevated at 0.73 mg/L (reference range: <0.5 mg/L). The cardiac troponin T level was elevated at 84 ng/l on admission, increased further to 1655 ng/l after 8 h and decreased to 607 ng/L at discharge (reference range: <15 ng/L). Serum pro-brain natriuretic peptide (pro-BNP) was also slightly elevated at 373 ng/L (reference range: <338 ng/L). The kidney function tests were normal with serum creatinine at 63 μmol/L (reference range: 45–90 μmol/L) and an eGFR of 96 mL/min/1.73 m^2^. 

**Figure 2:**
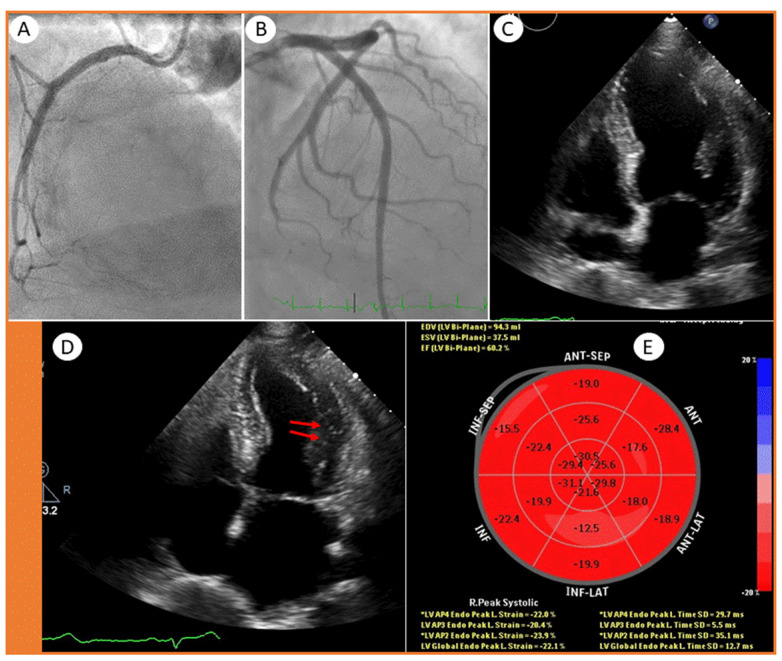
Coronary angiography showing a (**A**) normal right coronary artery and (**B**) normal left main stem, left circumflex artery with marginal branches and left anterior descending artery with diagonal branches. Echocardiographic 4-chamber views showing (**C**) end-diastolic and (**D**) end-systolic frames with mild hypokinesis (arrows), and a (**E**) Bulls’ eye plot.

Based upon symptoms including chest pain in combination with ECG changes and elevated cardiac biomarkers, the patient was put on standard NSTEMI treatment with acetylsalicylic acid, ticagrelor and a low molecular weight heparin, and a coronary angiography was scheduled. The coronary angiography revealed normal epicardial coronary arteries (Figure 2A,B). Due to a slightly elevated D-dimer, a chest CT-scan was performed. This did not show any signs of pulmonary embolism but minor lung opacities in regression (not shown).

A standard echocardiogram was repeated on the same day, which showed a normal LV ejection fraction of 60%. Similarly, the right ventricular function and size were normal, and there were no signs of valvular heart disease. However, on the 4-chamber view, there were 1–2 myocardial segments with hypokinesis in the mid-lateral wall (Figure 2C,D, arrows). Although the global longitudinal strain (GLS) was normal (−22%), the Bulls’ eye plot showed an impaired strain in the lateral/inferolateral segments (−12% and −17%) (Figure 2E), suggesting an ischemic or myocarditis-like injury of the left circumflex (LCx) artery supply region. 

The patient remained stable and did not experience further chest pain. She was continuously monitored with a telemetry and had a stable sinus rhythm. At this point, our working diagnosis was acute myocardial infarction, but myocarditis was a possible differential diagnosis.

**Figure 3:**
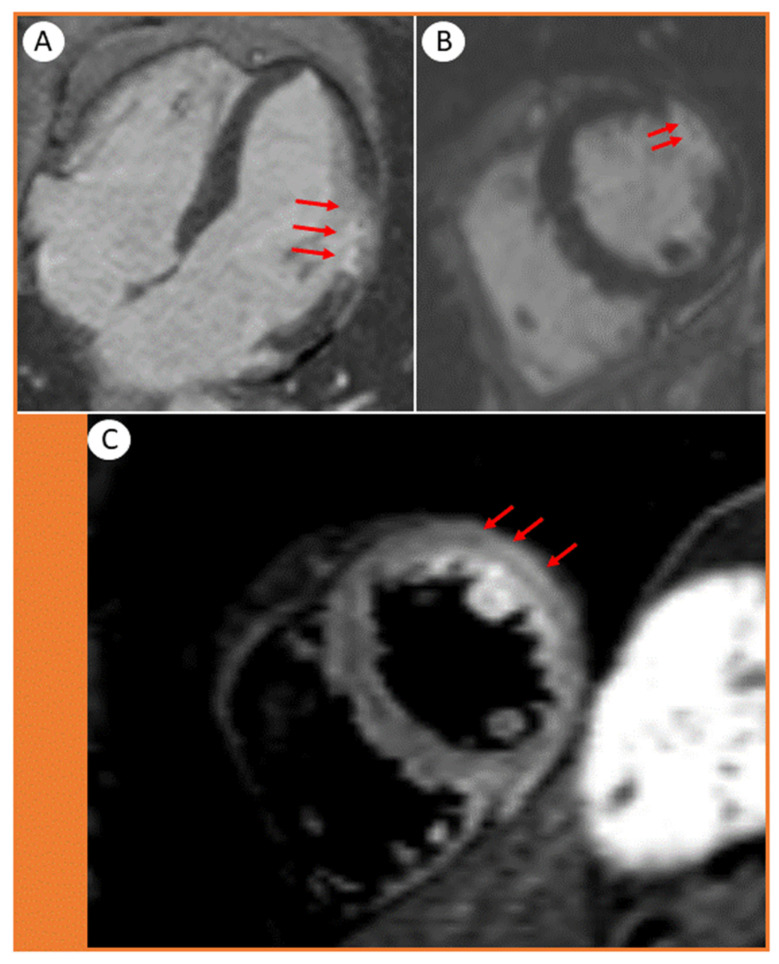
Cardiac magnetic resonance images showing (**A**) 4-chamber and (**B**,**C**) short axis views. The transmural distribution of late gadolinium enhancement (LGE) in the lateral wall of the left ventricle displays the extent of the infarction. (**B**, arrows) Microvascular obstructions are presented as tiny spots within the LGE. (**C**, arrows) The short-axis STIR acquisition shows edema in the lateral wall and papillary muscle.

Next, we performed a cardiac magnetic resonance (CMR), which showed transmural late gadolinium enhancement (LGE) in the mid-ventricular level of the lateral wall, involving the papillary muscle (Figure 3A–C, arrows). 

The findings were strongly suggestive of a transmural myocardial infarction/necrosis. The damaged area fitted well with obstruction in a minor myocardial branch of the LCx, most likely by a microthrombus (Figure 3B, arrows). The presence of edema in the same segments (Figure 3C, arrows) suggested that the infarction was relatively recent, also supported by the typical rise and fall pattern of cardiac troponins. 

She was mobilized in the ward, and after an uneventful stay for five days she was discharged in a stable condition. The ECG at discharge showed a regression of ST-T changes in anteroseptal leads (Figure 1, lower panel). Her medication at discharge included a double antiplatelet treatment for six months and a statin. 

## 3. Discussion

There are three learning points to this case study: (1) Nonhospitalized COVID-19 patients who are often younger and experience milder symptoms may develop cardiac injury during recovery from COVID-19 infection; (2) Minor myocardial infarctions/injuries may be present despite normal epicardial coronary arteries by conventional angiography and are easily revealed on CMR; (3) The global LV ejection fraction may be normal, but there may be subtle regional wall motion abnormalities and impaired myocardial strain in the affected segments, suggesting subclinical LV dysfunction and ischemia. 

COVID-19 is a highly hypercoagulable state and is associated with thrombotic events both on the arterial and venous sides of the systemic circulation [1,2,3,4,5,6]. Myocardial injury can be caused by either acute ischemic injury due to thrombosis in the epicardial coronary arteries or by microvascular obstruction due to microthrombi, and it is associated with an increased risk of mortality [1]. A number of mechanisms have been proposed, such as the direct viral invasion of the heart and vascular endothelium, vasculitis, an immune-and inflammation-induced systemic cytokine storm and activation of the coagulation system, increased blood viscosity, endothelial dysfunction and microvascular obstruction involving small myocardial branches of the coronary arteries; i.e., arterioles and capillaries [1].

Furthermore, a hyperactivated platelet phenotype has been detected in COVID-19 infection, which is associated with platelet-leukocytes formation and neutrophil extracellular traps, favoring a pro-thrombotic state with subsequent microvascular thrombi [1]. This scenario was clearly illustrated by the present case report. The epicardial coronary arteries appeared normal on conventional angiography, and the ECG did not indicate typical STEMI features, which would normally be expected following acute transmural infarcts secondary to the obstruction of the main coronary arteries. However, the CMR conveniently confirmed that the culprit lesion was a minor LCx branch occluded by microthrombi, leading to a segmental transmural infarct and necrosis without causing typical ST-segment elevation. It is also important to highlight that microthrombi can also be part of a spontaneously resolved thrombotic occlusion of the epicardial arteries. However, in some COVID-19 cases it may be difficult to exclude myocarditis. 

The pathophysiology of acute coronary syndromes in COVID-19 is often independent of the pre-existing traditional CV risk factors. Of note, our patient did not have the traditional CV risk factors, a family history of thrombosis or any known thrombophilia state. Hence, the microthrombi were most likely due to the COVID-19-associated dysregulation of the inflammation/immune response and a hypercoagulable state, which was still evident in the recovery phase. 

Recent autopsy studies on patients who died from COVID-19 have provided important insights on the presence, extent and etiology of cardiac injury [7,8]. In a study of 40 hearts from COVID-19 patients who died, Pellegrini et al. found signs of cardiac injury in 35% of the hearts as reflected by small areas of infarct and focal myocyte necrosis due to thrombi, primarily microthrombi in small transmural vessels. Further analyses of these thrombi showed that they were composed of more fibrin and terminal complement C5b-9, suggestive of an immune-mediated reaction. Of note, myocarditis and huge infarcts involving the territorial distribution of the individual major coronary arteries were rare [7].

The thrombi may be difficult to detect clinically. In our case, the global LV ejection fraction was normal, and the hypokinetic segment in the lateral wall was hardly appreciable. However, speckle tracking echocardiography, a more sensitive method for assessing subclinical myocardial dysfunction, showed segmental strain impairment and raised the suspicion of underlying myocardial injury in a minor area within the lateral wall. CMR is an excellent imaging modality to differentiate between myocarditis-like injury, acute myocardial infarctions and stress-cardiomyopathy [9]. In our case, the late gadolinium enhancement (LGE) features on CMR confirmed the pattern, exact localization (involving a small transmural branch) and extent of the acute cardiac injury. The transmural LGE appearance suggested acute myocardial infarction rather than myocarditis. 

The fact that thrombi can occur as a consequence of a COVID-19-associated altered inflammatory/immune response and a hypercoagulable state supports the use of prophylactic antithrombotic and anticoagulation medications, as well as statins on a somewhat more liberal basis, particularly in patients with diabetes [10]. In addition to their lipid-lowering properties, statins also have a pleiotropic effect, reducing systemic inflammation and avoiding endothelial damage, not only in hospitalized COVID-19 patients but also in those with a milder form of the disease who recovered at home. The prophylactic use of antithrombotic and anticoagulation medications in COVID-19 patients should be tested in future prospective studies. 

## Data Availability

Upon reasonable request, we may make the data available to any researcher for purposes of reproducing the results or replicating the procedure.
